# Elemental vs. phase composition of breast calcifications

**DOI:** 10.1038/s41598-017-00183-y

**Published:** 2017-03-09

**Authors:** Robert Scott, Catherine Kendall, Nicholas Stone, Keith Rogers

**Affiliations:** 1 Biophotonics Research Unit, Gloucestershire Royal Hospital, Gloucester, UK; 2 Cranfield Forensic Institute, Cranfield University, Shrivenham, UK; 3 Biomedical Physics Group, Physics and Astronomy, University of Exeter, Exeter, UK

## Abstract

Despite the importance of calcifications in early detection of breast cancer, and their suggested role in modulating breast cancer cell behaviour, very little detail is known about their chemical composition or how this relates to pathology. We measured the elemental composition of calcifications contained within histological sections of breast tissue biopsies, and related this to both crystallographic parameters measured previously in the same specimens, and to the histopathology report. The Ca:P ratio is of particular interest since this theoretically has potential as a non-invasive aid to diagnosis; this was found to lie in a narrow range similar to bone, with no significant difference between benign and malignant. The Mg:Ca ratio is also of interest due to the observed association of magnesium whitlockite with malignancy. The initially surprising inverse correlation found between whitlockite fraction and magnesium concentration can be explained by the location of the magnesium in calcified tissue. Sodium was also measured, and we discovered a substantial and significant difference in Na:Ca ratio in the apatite phase between benign and malignant specimens. This has potential for revealing malignant changes in the vicinity of a core needle biopsy.

## Introduction

This study extends our earlier work investigating the crystallographic properties of breast calcifications, and its relationship with pathology^1^. Elemental composition is of interest for two main reasons.

Firstly, variation of composition with pathology could in principle be used to improve diagnosis. The characteristics currently employed to distinguish suspicious from benign calcifications in screening mammography are based on radiographic appearance, such as size, shape, and distribution. Despite the existence of well-developed radiographic risk scoring systems based on these characteristics, such as BI-RADS^2^, the most recent published results from the NHS England screening programme show that only 21% women referred for further assessment following screening subsequently receive a diagnosis of cancer^3^. There is therefore a clear clinical need for improvement of the positive predictive value of mammography. If predictive aspects of the chemical composition could be determined radiographically and overlaid on a mammogram, that could give valuable extra information to assist the radiologist with assessment. It could be likened to viewing the calcifications present on a mammogram in colour rather than black and white. In theory, it is possible to differentiate between calcified tissues with a dissimilar calcium to phosphorus ratio using dual energy radiography, and this has been demonstrated *in-vivo* ^4^. In addition, a simulation study suggested that it is theoretically possible to use dual energy imaging to discriminate Type 1 (calcium oxalate) from the more common Type 2 (hydroxyapatite) calcifications in breast tissue^5^. The technological challenges for practical implementation are formidable, particularly for discrimination of more subtle changes in Ca:P ratio within Type 2 calcifications. However, advances in detector technology, such as energy resolving photon-counting detectors, give some cause for optimism. In order for these measurements to be clinically useful, there does need to be a detectable difference in elemental composition between calcifications in benign and malignant cases. A principal aim of this study was therefore to determine whether there is a sufficient variation in Ca:P ratio with pathology for this to have diagnostic potential.

Secondly, there is also increasing evidence that calcifications are not passive bystanders, but can play an active role in breast cancer progression^6–8^. The importance of calcifications as part of the breast cancer microenvironment is perhaps not surprising, given that calcified tissue forms fertile ‘soil’ for breast cancer metastasis, as was pointed out by Stephen Paget in 1889^9^. There is also evidence that breast cancer cell behaviour depends on the characteristics of the hydroxyapatite crystallites^10^. Despite this, current understanding of breast calcification chemistry is very sketchy.

Early studies on the composition of breast calcifications classified them into two distinct chemical and crystallographic types. “Type 1” calcifications are composed of calcium oxalate in the crystalline form of weddellite; these are comparatively uncommon, and are believed to be the result of benign processes. “Type 2” calcifications are composed of calcium phosphate with an apatite structure; these are much more common, and are sometimes, but not always, the result of malignancy. Subsequent work has shown that Type 2 calcifications are rather variable in composition and structure. Some aspects of this physico-chemical variability are related to pathology, and could be diagnostically useful. For instance, a strong correlation has been found between carbonate substitution and pathology classification^11, 12^, and this could in principle be probed non-invasively using Raman Spectroscopy^13^. Microstructure of other pathological calcifications has also been shown to have potential for determining aetiology^14^. Breast calcifications also exhibit microstructural variations, such as a spherulitic structure that appears be related to malignancy^15^.

There are theoretical reasons why the elemental composition of calcifications may differ between calcifications of benign and malignant origin. A very wide range of ions can be incorporated as substitutions within the hydroxyapatite lattice^16^. The extent of substitution, and hence the elemental composition, depend on the ionic environment as calcifications form. This is similar to the way that precipitated apatitic biomaterials with a controlled range of substitutions can be synthesised by varying reagent composition. There are several proposed initiation sites for the formation of breast calcifications, including apoptotic bodies, matrix vesicles, and extracellular matrix, or possibly a combination^8^. Both intracellular and extracellular ionic concentrations can be substantially altered in tumour tissue as a result of metabolic changes that affect ion transport across the cell membrane. For instance, elevated intracellular sodium and magnesium are associated with mitogenesis and markedly high levels are seen in tumour cells^17–20^. Breast tumour soft tissue has also been shown to be enriched in potassium, calcium, iron, copper, and zinc^21, 22^. It is unclear whether there are similar patterns of element enrichment and depletion in calcifications associated with tumours. Our earlier study showed a variation in crystallographic parameters of hydroxyapatite with pathology, and that malignancy is associated with the presence of increased quantities of magnesium whitlockite. These both provide indirect evidence for variation in elemental composition with pathology, which we sought to confirm by direct measurement of the same set of biopsy specimens.

Diagnostic archives offer ready access to a wide range of specimens, but there are few suitable techniques for analysis of the elemental composition of a calcification within a histological section. We chose to use Energy-dispersive X-ray spectroscopy in a scanning electron microscope (SEM-EDS), since it is well suited to measurement of major elements in calcified tissue of a few micrometres thick (typical of breast tissue histological sections). In addition, it offers excellent spatial resolution, and is readily available. X-ray fluorescence is an alternative, but has much poorer spatial resolution, and with calcified tissue of a few micrometres thick, the elemental signals are strongly dependent on thickness, which makes quantitative analysis difficult or impossible in specimens of uneven thickness over the irradiated area. It should be noted that quantitative analysis of this type of specimen by SEM-EDS does have many potential pitfalls, which are addressed in the methods section.

## Results

### Calcfication Morphology

**Figure 1:**
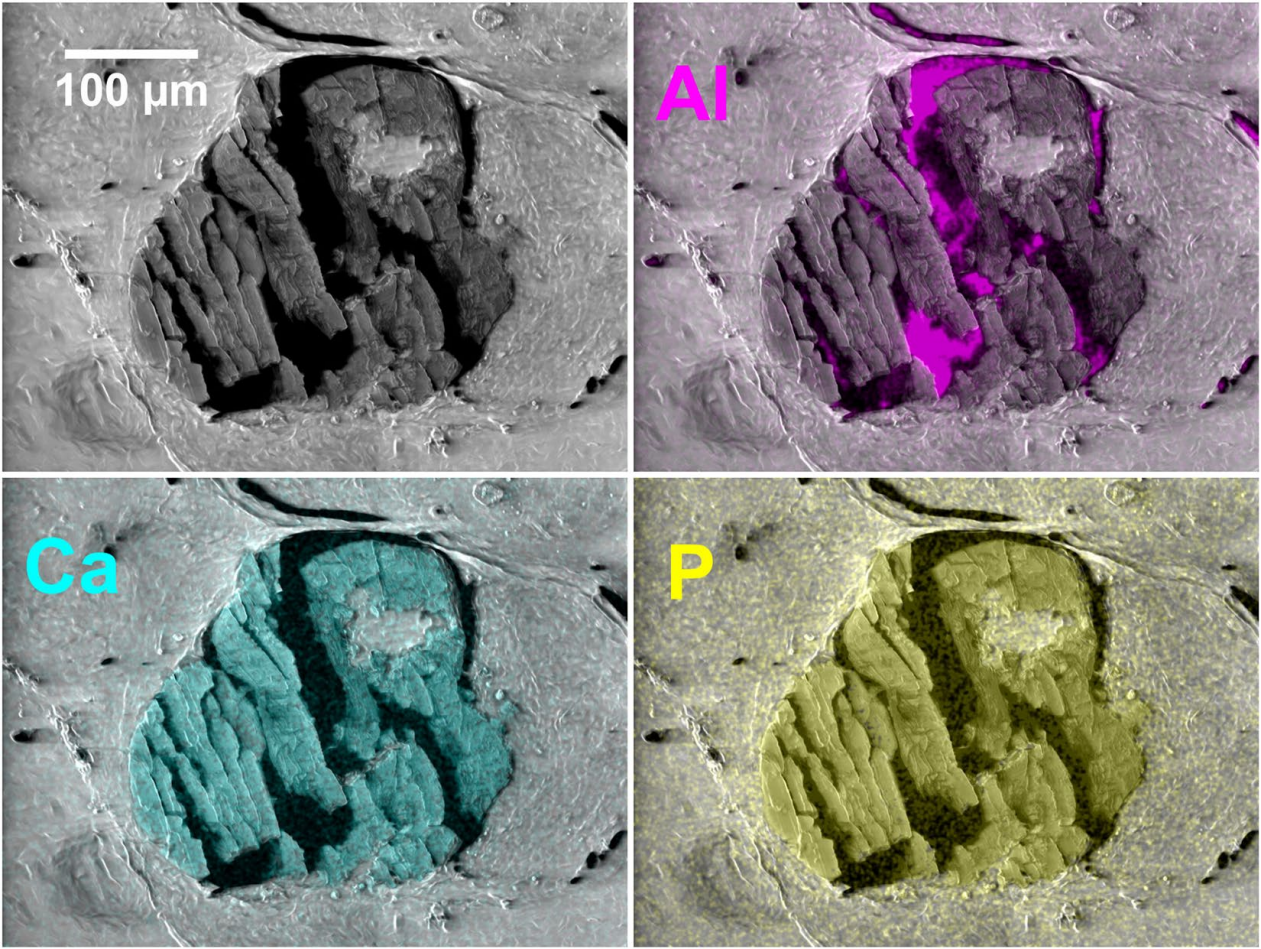
SEM image of a typical calcification overlaid with EDS elemental maps. The aluminium substrate can be seen through the cracks in the calcification resulting from sectioning. (Specimen S44 – pathology details in Supplementary Table A).

**Figure 2:**
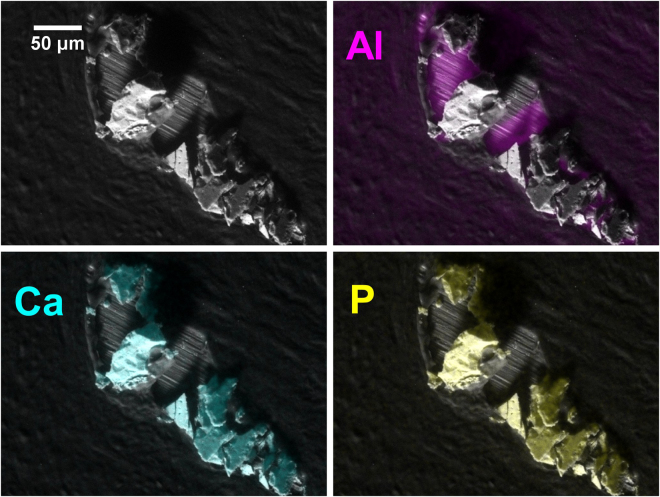
SEM image of a highly fragmented calcification. Note the shadowing of the substrate in the Al image. (Specimen S59 - pathology details in Supplementary Table A).

An SEM-EDS image of a typical calcification is shown in Fig. 1. Although the calcification has been fragmented by sectioning, it remains essentially complete. As discussed in the methods section, a few calcifications were extensively disrupted by the microtome blade. An example is shown in Fig. 2. These calcifications were generally characterised by a wider confidence interval for the median of measured composition.

### Calcium Phosphorus Ratio

**Figure 3:**
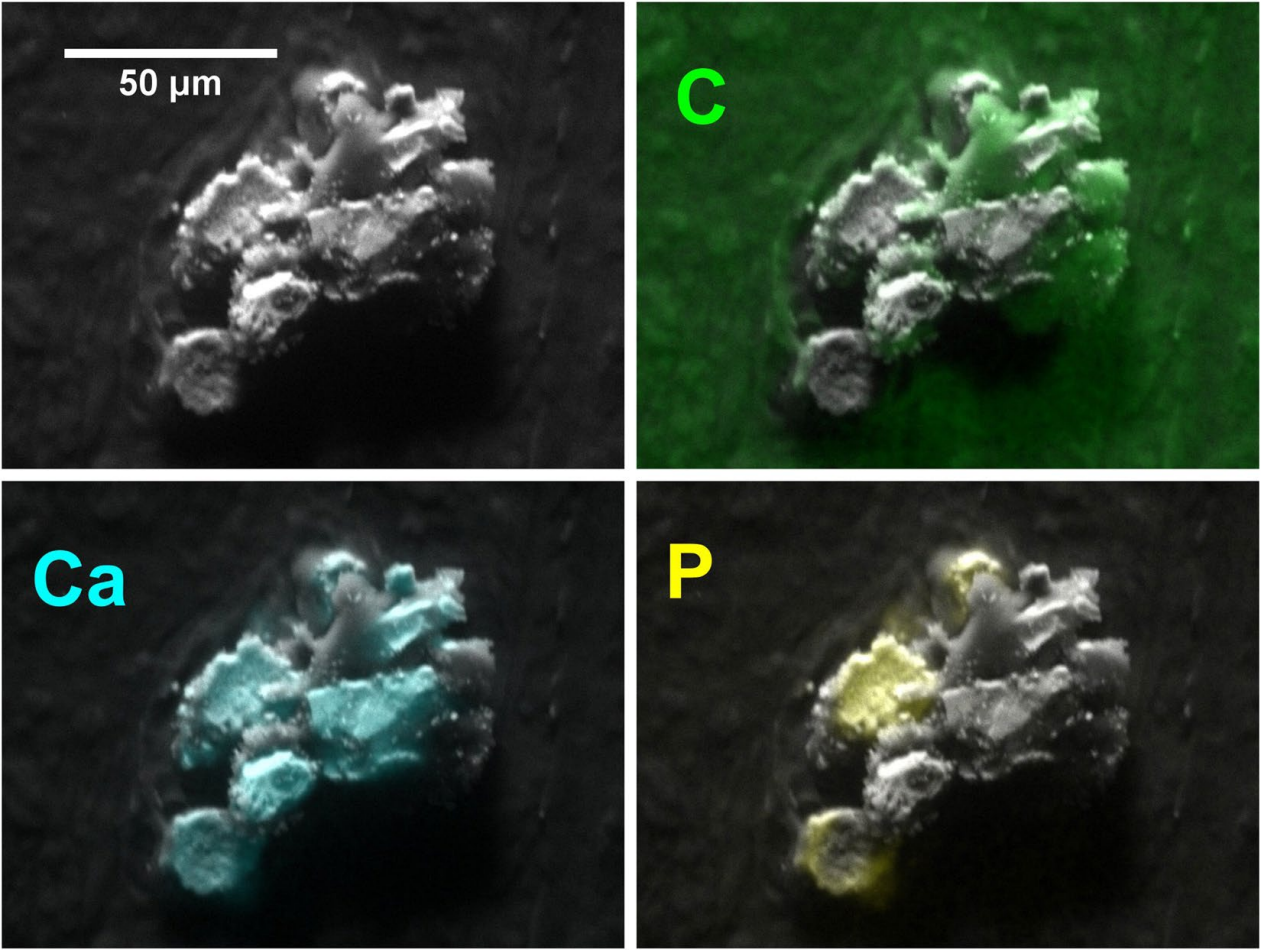
SEM image of a calcification with low phosphorus regions corresponding to a non-apatite phase. (Specimen S66 – pathology details in Supplementary Table A).

All bar one of the calcifications had a measured atomic Ca:P ratio in the range of 1.54 to 1.92, with a mean and median of 1.70. The one Ca:P outlier consisted of a small (~Ø70 μm) calcification in an invasive specimen, in which the Ca:P measurements fell into two very distinct groups: 15 point measurements had a measured Ca:P ratio >100, and 12 measurements had a Ca:P ratio in the normal range (median 1.78). The normal and low-phosphorus measurements were in discrete areas, as shown by the calcium and phosphorus maps in Fig. 3. Although an isolated occurrence, the measurements from this calcification proved important in interpreting the results with minor elements.

**Figure 4:**
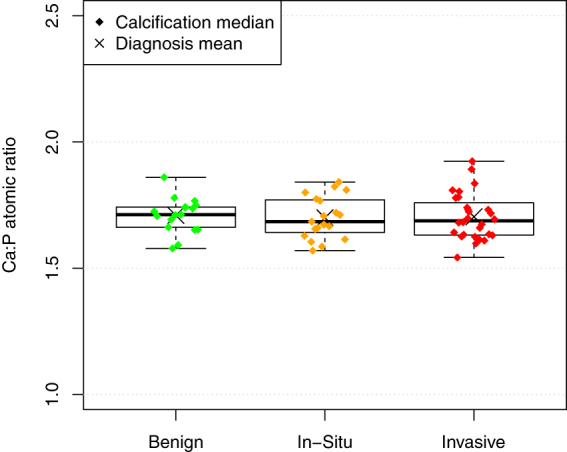
The Ca:P ratio of calcifications shows no systematic variation with specimen pathology.

The relationship between pathology and the Ca:P ratio, averaged by calcification, is shown in Fig. 4. The mean Ca:P ratio was 1.70, 1.69 and 1.70 for benign, *in-situ*, and invasive respectively. In addition, the apparent difference in variance between pathology groups was not found to be significant (Brown-Forsythe test).

### Magnesium Calcium Ratio

**Figure 5:**
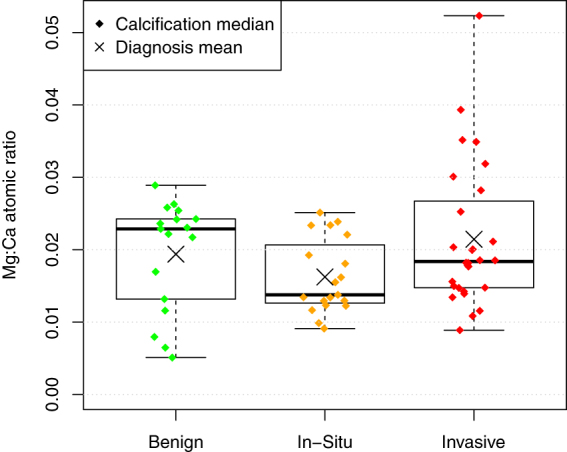
No significant difference in Mg:Ca ratio for calcifications was found between diagnoses.

The mean Mg:Ca atomic ratio was calculated as 1.93% (95% confidence intervaI 1.70–2.11%). The relationship between pathology and the Mg:Ca ratio, averaged by calcification, is shown in Fig. 5. Although the 6 highest Mg:Ca levels out of 66 calcifications were all in specimens with an invasive diagnosis, there was no significant difference between the pathology groups.

The single calcification with a very high Ca:P ratio showed a large difference in magnesium level between the low-P and high-P areas within the calcification. The 15 measurements from the low-P areas had a Mg:Ca ratio in the range 0.1–1.1% (mean 0.7%), whereas the 12 measurements from the high-P areas had a Mg:Ca ratio in the range 1.8–2.7% (mean 2.2%).

### Sodium Calcium Ratio

**Figure 6:**
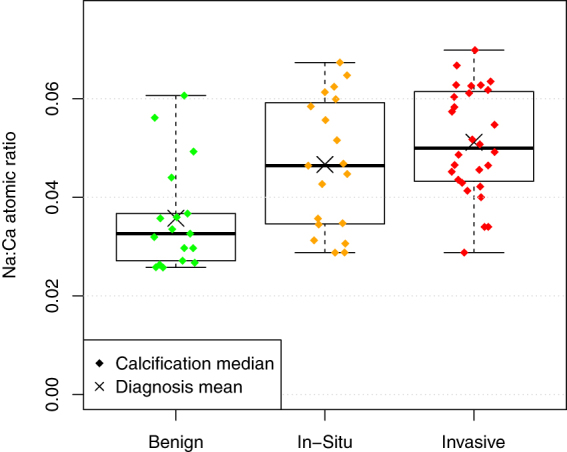
The Na:Ca ratio of calcifications in specimens with a benign diagnosis is significantly lower than that in specimens categorised as malignant (In-Situ or Invasive).

The relationship between pathology and the Na:Ca ratio, averaged by calcification, is shown in Fig. 6. Unlike the other two elemental ratios, there is a highly significant difference between groups. Treating diagnosis as an ordinal variable, ranked benign < *in-situ* < invasive, the Na:Ca ratio is positively correlated with diagnosis (p < 0.001 Kendall's tau-b). With the measurements aggregated to a mean specimen level, the significance of the correlation becomes p = 0.007. If diagnosis is treated as a binary variable, mean specimen sodium level is significantly lower in benign than malignant specimens (p = 0.007 Mann-Whitney U).

Measurements of sodium levels need to be treated with caution. The specimens are immersed in saline solutions during fixation, hence at least some of the sodium present might not originate from the calcification. However, the single calcification with areas of low-phosphorus calcified material provides evidence that the sodium detected is intrinsic to the apatite phase. As with magnesium, the sodium measurements fell into two very distinct groups. The 15 measurements from the low-P areas had a low sodium content, with a Na:Ca ratio in the range 0.2–1.5% (mean 0.8%). In contrast, the 12 measurements from the high-P areas had high Na:Ca ratio in the range 3.3% to 6.4% (mean 5.1%). As confirmation that this is not an artefact in which the signal from all low atomic number elements is suppressed in the low phosphorus areas, or that an inadequately corrected oxygen sum peak is masquerading as sodium, the mean weight percentage of oxygen was 49% and 48% in the low and high phosphorus regions respectively.

### Other Elements

Other elements of interest are potassium, iron, and zinc, since elevated levels of these elements have been observed in breast tumour tissue^23, 24^. Levels of these elements within the calcifications in this study were all below detection levels. The absence of potassium is not surprising since K^+^ substitution for Ca^2+^ in apatite is much less energetically favourable than sodium, due the relatively large size of the potassium ion^25^. This is consistent with the observation that potassium levels in bone are an order of magnitude less than sodium^26^. Zinc levels in bone are a further order of magnitude less than potassium^26^, and the four-fold enhancement observed in tumour tissue^21, 24^, to about 7–12 ppm, is unlikely to raise levels in calcifications to a level detectable by SEM-EDS. Increased iron levels in tumours are very likely due to increased vascularity, as with zinc, the levels in bone and tumour soft tissue are far too small to make this a likely candidate for detection in this study.

### Correlation with X-Ray Diffraction Data

The three elemental ratios measured were compared to crystallographic parameters previously measured^1^ in the 39 calcifications for which both sets of measurements were available, and are shown in Table 1. The lattice parameter ‘c’/‘a’ ratio was used since the ‘a’ and ‘c’ parameters are strongly negatively correlated in these specimens. Linear regression was conducted with XRD measurements as a function of EDS measurements. The p-value for a correlation was adjusted to control the familywise error rate for multiple comparisons using the Holm–Bonferroni method. After adjustment, the only significant correlations between crystallographic parameters and composition relate to the magnesium calcium ratio. Although the correlations are all weak, domain size, c/a lattice parameter ratio and whitlockite percentage were all significantly negatively correlated with magnesium concentration.

**Table 1:** Correlation of elemental ratios measured in this study, with X-ray diffraction measurements made on the same 39 calcifications in a previous study.

EDS Measurement	XRD measurement	Coefficient	Adjusted R^2^	p-value	Adjusted p-value
Mg:Ca Ratio	Whitlockite %	−35.6	0.183	0.004	**0.034**
	Domain Diameter	−125	0.165	0.007	**0.046**
	‘c’/‘a’ lattice parameter ratio	−0.105	0.279	<0.001	**0.003**
Ca:P Ratio	Whitlockite %	2.73	0.098	0.032	0.158
	Domain Diameter	−0.0555	−0.028	0.991	1.000
	‘c’/‘a’ lattice parameter ratio	0.00231	−0.012	0.459	1.000
Na:Ca Ratio	Whitlockite %	−1.29	−0.027	0.871	1.000
	Domain Diameter	63.1	0.107	0.026	0.154
	‘c’/‘a’ lattice parameter ratio	0.0366	0.071	0.056	0.222

## Discussion

### Calcium Phosphorus ratio

The calcium to phosphorus ratio was of primary interest in this study, since if there were a substantial variation with pathology, it would open up the possibility of additional diagnostically useful information from mammography using energy discriminating detectors. However, the measured Ca:P ratio of calcifications found to lie in a narrow range (interquartile range 1.64 to 1.75), and no significant difference could be found between the pathology groups. These results do not preclude the possibility that some diagnostically useful relationship may exist within a particular sub-type of calcifications, but the apparent lack of a general relationship limits the appeal of this approach.

The Ca:P ratio in breast calcifications has been measured previously in one small study using SEM-WDS (Wavelength Dispersive Spectroscopy)^27^. This involved three benign and two malignant specimens, with 2–5 calcified tissue spectra collected per specimen. The overall mean molar Ca:P ratios for benign and malignant specimens were 1.64 and 1.67 respectively (weight ratios 2.13 and 2.17), which is very similar to our study.

It has been suggested that the mechanisms involved in the formation of breast calcifications may be similar to those involved in the deposition of hydroxyapatite in bone; in particular, bone matrix proteins involved in osteoblast mineralisation are also expressed in mammary cells^28^. Measured values for Ca:P ratio in bone vary widely, and have recently been comprehensively reviewed^26^. The most relevant all-bone study cited is the ICRP-89 Reference Woman with a Ca:P atomic ratio of 1.63, and a compilation of studies on various bones gave a value of 1.68. These are very similar to the values found for breast calcifications in this study. Calcifications appear to be similar to bone in Ca:P ratio as well as in crystallographic parameters.

The low phosphorus areas in one calcification must consist of a non-apatite phase. The most likely contender is calcium oxalate, though without evidence from x-ray diffraction and/or vibrational spectroscopy, this cannot be confirmed. Although most calcium oxalate crystals have been reported in association with benign lesions, they have also been observed in malignant cases, where they are presumed to be bystanders^29^. It is also notable that this instance occurs within a calcification which also contains regions of hydroxyapatite, whereas there are no previous reports of a mixed Type I/II calcification.

### Magnesium Calcium ratio

We previously reported an increased level of magnesium whitlockite in malignant specimens compared to benign. We therefore expected to find higher levels of magnesium in the malignant specimens. Not only did we find no such correlation between magnesium levels and pathology, but we found a statistically significant *inverse* correlation between Mg:Ca ratio and percentage of magnesium whitlockite, as determined by x-ray diffraction.

This initially surprising finding can be explained from the nature of magnesium within calcified tissue. Magnesium in bone resides largely on the crystal surface^30, 31^ or hydration layer^32, 33^ rather than within the apatite lattice. Apatite has been shown to be capable of only very limited magnesium substitution under physiological conditions^16^. Much of the magnesium in calcified tissue can therefore exchange rapidly, and forms the main reservoir for buffering extracellular magnesium in the body^34, 35^. Extracellular levels of magnesium have been observed to be, on average, lower than normal in breast cancer^36^. Even if a calcification initially forms with high levels of magnesium, due to high intracellular levels, magnesium associated with hydroxyapatite could rapidly decrease towards the lower levels present in the extracellular environment. This is particularly likely given the high surface area to volume ratio of a particle the size of a breast calcification. It is also conceivable that at least some labile magnesium could leach from calcifications within the specimen through immersion during fixation and histological processing.

In contrast to the apatite phase, the magnesium in whitlockite forms an essential stabilising element of the crystal lattice^37, 38^. The presence of whitlockite is therefore an indicator of the local magnesium concentration at the time of mineralisation, rather than the time of sampling^39^. In precipitation experiments where there are both carbonate and magnesium ions in solution, it has also been observed that lower pH leads to less magnesium incorporation into the apatite lattice for a given magnesium concentration in the solution^40^. Furthermore, at lower pH, the appearance of whitlockite occurs at lower magnesium concentrations^40^. The pH is, on average, likely to be lower in malignant specimens than benign, thus inhibiting incorporation of magnesium into the apatite lattice, and instead favouring whitlockite formation. This offers an explanation for the increased whitlockite fraction in malignant specimens. The reported high intracellular^41^ vs. low extracellular^36^ levels of magnesium in breast cancer would resolve the apparent contradiction that whitlockite percentage appears to be negatively correlated with magnesium content.

Note that high intracellular levels of magnesium could still explain the low levels of carbonate observed in calcifications associated with tumours, as previously suggested^1^, since the carbonate level within the apatite lattice is influenced by the magnesium concentration at the time of crystallisation.

### Sodium

One of the most interesting and potentially useful observations in this study is the presence of significantly higher sodium levels in calcifications within malignant specimens. This is consistent with the observation that total tissue sodium levels are elevated within breast tumours^42, 43^. Intracellular levels of sodium are known to be elevated within tumour cells^17, 18^, whereas it is postulated that the extracellular sodium concentration remains relatively constant, provided there is exchange with the blood pool^42^. This suggests a predominantly intracellular origin for the calcifications observed in this study. Sodium readily substitutes for calcium in hydroxyapatite, and the level of substitution is likely to reflect the sodium concentration at the time of apatite precipitation. It appears that this difference survives histological processing, due to the fact that the sodium is substituted within the apatite lattice. If it can be confirmed that all calcifications arising from malignant changes display elevated sodium levels, this has potential to indicate malignancy in the vicinity of a needle biopsy specimen, even if malignant cells are not contained within the core.

## Materials and Methods

### Tissue Specimens

This study consisted of further characterisation of the specimens described in a previous publication^1^, together with additional specimens of the same type. These consisted of Formalin Fixed Paraffin Embedded (FFPE) core biopsy breast specimens selected from the Gloucestershire Hospitals NHS Foundation Trust diagnostic archive. The study was approved by Gloucestershire Local Research Ethics Committee and all methods were performed in accordance with relevant guidelines and regulations. Blocks were randomly selected from the 2012 archive, subject to the presence of calcifications in the histopathology report, and an unambiguous classification of “B2 - Benign”, “B5a - Ductal Carcinoma *In-Situ*”, or“B5b - Invasive Carcinoma”^44^. For the purposes of analysis, specimens were also grouped as “Benign” (B2) vs. “Malignant” (B5a or B5b). A summary of the histopathology report for each of the specimens analysed is given in Supplementary Information Table A. Blocks with radiographic evidence of calcifications near the cut surface were CT-scanned, and the reconstructed volumes processed as previously described^45^ to identify the position of calcifications relative to the tissue outlines at the surface of the blocks. A 5 μm slice from each block was mounted on a polished Ø25 mm aluminium alloy SEM stub.

### X-Ray Microanalysis

Elemental composition was measured using Energy-dispersive X-ray spectroscopy (EDS) in a Philips XL30 ESEM with an Oxford Instruments SiLi detector. The working distance was 12 mm, and approximately 200,000 total counts were taken per point measurement. A total of 2,789 spectra were collected from 66 calcifications in 31 specimens. Composition was calculated in AZtec 2.1 (Oxford Instruments). Measurement of composition in this type of specimen presents several challenges, which must be addressed in order to obtain reproducible results.

#### Charging

It was initially hoped that a 5 μm thick histological section in intimate contact with a polished aluminium SEM stub could be analysed under high vacuum without specimen charging, and indeed this proved to be the case with wax embedded soft tissue. However, the calcifications were fragmented by the microtome blade, frequently resulting in elevated flakes with only an edge in contact with the substrate. As a result, severe charging was observed with many calcifications. This could not be eliminated by carbon coating, due to the fragmented nature of the surface. Charging was eliminated by operating the SEM in environmental mode, with water vapour pressure 93 Pa (0.7 Torr). This also removed the need for carbon coating. Absence of charging was confirmed from observation of the Duane-Hunt bremsstrahlung limit. One potential bias introduced by operating in environmental mode is scattering of the beam, and consequently contamination of point measurements with signals from the ‘skirt’ of scattered electrons. With a working distance of 12 mm and a 20 kV accelerating voltage, only about 57% of the electron beam remains unscattered (scattering less than 10^−6^ Rad)^46^. In principle, therefore, measurements of a calcification could include a substantial contribution from surrounding soft tissue. To assess the magnitude of this effect, the typical contribution from soft tissue in biopsy cores containing calcifications was measured. Under identical collection conditions, soft tissue counts for phosphorus and calcium were approximately 2 and 3 orders of magnitude (respectively) lower than the counts from a calcification, and magnesium was below the level of detection. Theoretically, therefore, the Ca:P ratio could be underestimated by up to ~0.4% in a very small calcification as a result of the contribution from the skirt. In practice, this is substantially lower than other sources of variation for these measurements, hence can be disregarded. For verification, measurements on a small (~Ø10 μm) unfragmented calcification showed no significant variation in the Ca:P and Mg:Ca ratios with pressure over a range of 40–130 Pa (0.3–1.0 Torr).

#### Specimen Thickness

If the electron beam penetrates through the specimen to the substrate, this complicates the calculation of elemental concentrations, particularly if the local thickness varies within the skirt region of gas-scattered electrons. This difficulty can be eliminated if the combination of specimen thickness and beam energy ensures minimal beam penetration to the substrate. Using cortical bone as an analogue for the calcification matrix, the electron penetration depth of an unscattered 20 kV beam into a 5 μm thick slice was calculated using winCasino v2.48^47^. Elemental composition and density of cortical bone were taken from a NIST database^48^. The ϕ(ρ, z) curve shows that at 20 kV and slice thickness of 5 μm there is negligible penetration of electrons to the substrate. A straightforward bulk analysis calculation can therefore be performed.

#### Topography

The chemical composition within breast calcifications has been found to be very inhomogenous^15^, hence an area measurement over the whole cut surface of a calcification is an attractive option for measuring the average composition. However, as a result of fragmentation by the microtome blade, the calcification surface is not smooth and perpendicular to the beam. The local orientation of the surface relative to the electron beam and the detector can have a very substantial effect on the measured elemental concentrations. Moreover, this effect is not symmetrical; the disproportionate reduction in low energy x-rays from areas tilted away from the detector is not compensated by the areas oriented towards the detector, hence simple area averaging over the whole calcification will underestimate the concentration of low atomic number elements. The reduction in overall count rate from the areas tilted away from the detector mitigates this effect, but does not eliminate it. There are several strategies that can be adopted to correct for these topographic errors.

One option is to measure the peak to local background ratio. Since the background signal originates from the same electron interaction volume as the characteristic x-rays, it will be subject to the same absorption effects, and hence can be used to correct approximately for topographic errors. A practical problem with this approach is that the peak to background ratio in this measurement configuration is typically large (50:1 for calcium in this case) thus an excessively long collection time is needed to acquire sufficient background signal to calculate an adequately precise peak to background ratio.

Another approach is to take repeat point measurements in multiple specimen orientations, either by rotating the specimen around the beam axis, or with the use of multiple detectors. A simpler alternative to obtaining an average composition for the calcification involves measurement of multiple points over the calcification surface; if the orientation of measurement points in the beam-specimen-detector plane is symmetrically distributed relative the specimen normal, then the median measurement will approximate to the median composition with a surface orientation normal to the beam axis. This was verified with measurements distributed over the surface of a 70 μm spherical particle of synthetic hydroxyapatite. In some cases there was evidence of systematic orientation of flakes comprising the calcification, due to the directional cutting action of the microtome blade. A hybrid approach was therefore adopted, consisting of measuring multiple points, rotating the specimen by 180°, and measuring another set of points. The median and confidence interval of the elemental ratios of interest was calculated for the combined set of points using Efron’s nonparametric bias-corrected and accelerated (BCa) bootstrap method in R^49, 50^. Based on the 95% confidence interval, the mean precision of the median for a set of measurements on a calcification was ±3.4% for Ca:P ratio, and ±16% for both Mg:Ca and Na:Ca ratios.

## Electronic supplementary material

Supplementary Information Table A
