# Supplementary material for: Elemental vs. phase composition of breast calcifications
Source: Sci Rep. 2017 Mar 9;7:136. doi: 10.1038/s41598-017-00183-y (PMC5427875; doi:10.1038/s41598-017-00183-y)
Supplement: Supplementary file 1 — Supplementary Information Table A [file 41598_2017_183_MOESM1_ESM.pdf]

Elemental vs. phase composition of breast calcifications

Robert Scott, Nicholas Stone, Catherine Kendall, Keith Rogers

Supplementary Information – Table A

Note: Specimen numbering is the same is the previously published x-ray diffraction study, but the prefix S (instead of X) denotes that the section is mounted on an SEM stub.

**Specimen**	**Histological Opinion**	**Micro. Report: Summary extract**
S43	B5a	All three cores contain intermediate and high nuclear grade solid-type ductal carcinoma in-situ (DCIS) associated with some comedo necrosis and malignant microcalcification. There is cancerisation of lobules also present. There are some areas concerning for invasive malignancy but a definitive diagnosis cannot be made at this time. Immunohistochemistry will be carried out to see if this can be taken any further. At this time, the most appropriate categorisation is one of B5a.<br>Immunohistochemistry has been carried out and the areas of DCIS show rimming by myoepithelial cells. There is no definitive diagnosis of invasion and therefore the categorisation of B5a stands. Oestrogen receptor shows the DCIS to be strongly oestrogen receptor positive giving a quick score, if appropriate, of 8/8.
S44	B2	Fibroadipose breast tissue including a fibroadenoma. Dystrophic calcification is noted within the fibroadenoma. There is no cytological atypia.
S45	B5a	Intermediate and high nuclear grade DCIS associated with malignant microcalcification. DCIS shows solid cribriform and papillary growth patterns. There is no evidence of invasive malignancy.
S46	B5b	High nuclear grade DCIS associated with malignant microcalcification and invasive ductal carcinoma which appears to be moderately differentiated. Immunohistochemistry for oestrogen receptors demonstrates that more than 67% of neoplastic cells of this tumour show strong nuclear positivity, giving a Quick score of 8/8.
S47	B5b	These fragments of breast tissue bear infiltrating carcinoma, on this evidence moderately differentiated ductal carcinoma comprising cohesive nests and cords of cells infiltrating hyalinised fibroelastotic stroma. The tumour does show some lobular features. In addition there is high nuclear grade DCIS of solid and cribriform types with comedo necrosis and associated malignant calcification. Immunostaining for oestrogen receptors shows the tumour to be strongly ER positive (quick score 8/8). The tumour is E-Cadherin positive supporting the diagnosis of ductal carcinoma.
S48	B2	These biopsies are of breast tissue showing dense stromal sclerosis associated with coarse calcification which appears dystrophic. The epithelial elements show columnar cell change and mild usual type hyperplasia. There is no evidence of atypia or of in-situ or invasive malignancy in these biopsies.
S49	B5a	These breast core biopsies show intermediate nuclear grade ductal carcinoma in-situ of cribriform type with apocrine features. There is associated malignant microcalcification. There is no evidence of invasion in these biopsies.
S50	B5b	Cores of sclerotic and elastotic stroma which are infiltrated by a ductal carcinoma. The tumour appears well differentiated with well

		formed ducts lined by bland epithelial cells. There is much micro-calcification associated with the lesion and a small in-situ component is also present. Immunohistochemistry shows strong nuclear positivity in almost all tumour cells for oestrogen receptor giving a Quick score of 8/8.
S51	B2	The specimen comprises rather fragmented cores of benign breast tissue exhibiting a normal tubulo-lobular architecture with quite marked hyaline fibrosis of the stroma. There is some evidence of fibrosis of the intra-lobular stroma suggesting some fibrocystic change but some of the fragments show fibroadenomatoid features comprising expanded hyaline hypocellular stroma within which, there are some curvilinear compressed ducts. This fibroadenomatoid tissue is associated with large foci of micro-calcification. Although appearances may represent fibroadenomatoid change, part of a fibroadenoma is also a possibility. There is no evidence of malignancy.
S52	B5b	These core biopsies are infiltrated by a well differentiated ductal carcinoma (T1 P2 M2). There is associated microcalcification and focal DCIS. Immunohistochemistry for oestrogen receptors demonstrates that more than 67% of neoplastic cells of this tumour show strong nuclear positivity, giving a Quick score of 8/8.
S53	B5a	Histological assessment reveals that both specimens consist of cores of breast tissue within which there is high nuclear grade ductal carcinoma in-situ with a solid growth pattern associated with comedo necrosis and malignant micro-calcification. In areas, this has the features more in keeping with pleomorphic lobular carcinoma in-situ (LCIS) but, from a management point of view, should be regarded as DCIS. There is a some scarring and inflammation associated with some of the ducts but there is no definite evidence of invasive malignancy, although, in specimen 2, is this more concerning and immunohistochemistry will be carried out and a supplementary report will be issued. SUPPLEMENTARY: Immunohistochemistry has been carried out. There is no evidence of invasive malignancy. The in-situ component is negative for E-cadherin. This is considered to pleomorphic lobular carcinoma in-situ (LCIS). From a management point of view, it should be treated as for DCIS. The categorisation of B5a remains.
S54	B5b	A core of desmoplastic fibro-elastotic stroma bearing infiltrating adenocarcinoma, on this evidence moderate to poorly differentiated ductal carcinoma. Some probable high nuclear grade DCIS showing comedo necrosis is seen and there is malignant calcification. Immunostaining for oestrogen receptors has been performed (1) revealing the tumour to be strongly ER positive (quick score 8/8).
S55	B2	Stereo-cores right breast, representative calcs seen: This specimen comprises benign breast tissue exhibiting a normal tubulo-lobular architecture. There are well established fibrocystic changes with areas of fibrosis of the inter and intra-lobular stroma with dilatation and separation of acini. Apocrine and lactational type change is seen and in some areas, there is columnar cell change associated with benign micro-calcification. There is some cross cutting of the ducts. The specimen has been examined on multiple levels and neither in-situ nor infiltrating neoplasia is seen.
S56	B5b	This core biopsy shows a grade 1 infiltrating ductal carcinoma, not otherwise specified (tubules 1, pleomorphism 2, mitosis 1). Malignant microcalcifications are seen. There is no convincing DCIS. ER Quick score is 7/8 (5 + 2).
S57	B5a	1 and 2: Biopsies from both sites consist of breast tissue showing similar features with intermediate nuclear grade ductal carcinoma in-situ showing apocrine features. There is associated comedo necrosis with malignant microcalcification. There is no evidence of invasive malignancy in either specimen.
S58	B2	These core biopsies show features of fibrocystic change with many areas of sclerosing adenosis and focal benign microcalcification. There is no evidence of epithelial hyperplasia, in situ or invasive malignancy.
S59	B5b	Contains infiltrating ductal carcinoma, poorly differentiated, Grade 3 (tubules 3, pleomorphism 3, mitoses 2). There is no definite in-

		situ component but there is both necrosis and malignant calcification present. ER quick score of 7/8.
S62	B5b	Comprises cores of breast tissue bearing infiltrating carcinoma, on this evidence moderately differentiated ductal carcinoma. The parenchyma bears a chronic inflammatory infiltrate and there is an intra-ductal flat and largely columnar cell proliferation showing variable atypia that in areas is considered to represent flat intermediate to high grade DCIS. Stromal micro-calcification is also seen. Immunostaining for oestrogen receptors shows the tumour to be ER positive (Quick score 7/8).
S63	B5b	A core of fibroadipose breast tissue. There is marked stromal sclerosis within which there are breast structures that show some cystic change. Microcalcifiction is seen. One of the ducts is markedly dilated and another one shows columnar cell change. Within the sclerotic stroma there are two or three epithelial profiles with nuclear enlargement. This develops into a small focus of invasive ductal carcinoma on the deeper levels. Approximately half of the tumour cell nuclei are strongly positive for oestrogen receptors. Quick score 7/8.
S64	B2	Cores of breast tissue showing fibrocystic change with some cystically dilated ducts, others that show columnar cell change and a stroma that shows hyalinosis in places. Benign microcalcification is seen in some of the dilated ducts.
S65	B5a	These breast core biopsies show high nuclear grade ductal carcinoma in-situ of solid type with comedo necrosis and malignant microcalcification. There is no evidence of invasive malignancy.
S66	B5b	These are cores of fibro-adipose connective tissue which show widespread infiltration by a poorly differentiated carcinoma with ductal morphology. In this material the tumour appears to be grade 3 (T3 P3 M2). No conspicuous in-situ component is noted.Oestrogen receptor status is very weakly positive with only a small proportion of cells showing weak staining (Quick score 2/8).
S67	B2	Tissue fragments comprising haemorrhagic adipose tissue together with breast tissue showing sclerotic stroma within which are benign ducts and lobules. Calcified material is seen associated with sclerotic stroma. A small amount of epidermis is also present. No other lesion is identified. Some dilated ducts are present on deeper levels and the appearances raise the possibility of sampling from an area of fibrocystic change. There is some calcification associated with these cystically dilated ducts. The appearances in this biopsy can be regarded as benign.
S68	B5b	Cores of breast parenchyma bearing focally necrotic infiltrating carcinoma, on this evidence poorly differentiated ductal carcinoma. There is high nuclear grade DCIS of micropapillary type with comedo necrosis. Immuno staining for oestrogen receptors shows the tumour to be strongly ER positive (Quick score 8/8).
S69	B5a	Intermediate nuclear grade ductal carcinoma in-situ showing apocrine features. There is associated comedo necrosis with malignant microcalcification. There is no evidence of invasive malignancy.
S70	B5b	These core biopsies are infiltrated by a tumour composed of angulated tubules set in a desmoplastic stroma. This tumour is likely to be a grade 1 tubular carcinoma (tubules 1, pleomorphism 2, mitosis 1). Malignant micro-calcifications are seen. There is no DCIS. Oestrogen receptor immunohistochemistry shows strong nuclear staining in all tumour cells. ER Quick score is 8/8 (5+3).
S71	B2	Fragmented pieces of fibrofatty breast tissue plus some clot. There is evidence of fibrocystic change and columnar cell change and in one area, an aggregate of slightly ectatic separated acini are associated with benign calcification. Although one duct, in the most superficial levels shows some architectural complexity, this is considered an artefact due to cross cutting. The stromal collagen appears slightly fibrillary in areas but established elastosis is not seen.

		Neither in-situ nor infiltrating neoplasia is identified and a B2 diagnosis is considered appropriate.
S72	B5b	These core biopsies are widely infiltrated by a moderately differentiated ductal carcinoma exhibiting modified Bloom Richardson grade 2 features (T2 P2 M2). The tumour is associated with malignant microcalcification. There is no in-situ component identified in these biopsies. Immunohistochemistry shows strong nuclear positivity in almost all tumour cells for oestrogen receptor, giving a Quick score of 8/8.
S73	B5a	These core biopsies show foci of ductal carcinoma-in-situ exhibiting central comedo necrosis and malignant microcalcification. DCIS shows intermediate nuclear grade features. No evidence of invasive malignancy is seen in these core biopsies.
S74	B2	These breast core biopsies show dense stromal sclerosis with benign microcalcification and some features on deeper levels of previous fat necrosis. The deepest level includes a cluster of cells within the fat which may be macrophages in association with the fat necrosis. Immunohistochemistry has been performed and ovarall histological appearances are regarded as those of fibrocystic change with possible minimal focal fat necrosis and no atypical or neoplastic features.
S75	B5b	Cores contain infiltrating carcinoma which, on this evidence, is a classical lobular carcinoma which is moderately differentiated equating to a modified Bloom and Richardson grade of grade 2 (tubules 3, pleomorphism 2, mitoses 1) and is associated with lobular carcinoma in situ (LCIS) and malignant microcalcification. Immunohistochemistry for oestrogen receptor shows moderate and focally strong staining in the vast majority of infiltrating carcinoma cells giving a Quick score of 7/8.
